# Protein Polymer-Based Nanoparticles: Fabrication and Medical Applications

**DOI:** 10.3390/ijms19061717

**Published:** 2018-06-09

**Authors:** Kelsey DeFrates, Theodore Markiewicz, Pamela Gallo, Aaron Rack, Aubrie Weyhmiller, Brandon Jarmusik, Xiao Hu

**Affiliations:** 1 Department of Physics and Astronomy, Rowan University, Glassboro, NJ 08028, USA; defratesk6@students.rowan.edu (K.D.); weyhmilla9@students.rowan.edu (A.W.); jarmusikb1@students.rowan.edu (B.J.); 2 Department of Biomedical Engineering, Rowan University, Glassboro, NJ 08028, USA; markiewit3@students.rowan.edu; 3 Department of Molecular and Cellular Biosciences, Rowan University, Glassboro, NJ 08028, USA; gallop3@students.rowan.edu (P.G.); racka9@students.rowan.edu (A.R.)

**Keywords:** protein, nanoparticles, biomaterials fabrication, nanomedicine, bioimaging, drug delivery

## Abstract

Nanoparticles are particles that range in size from about 1–1000 nanometers in diameter, about one thousand times smaller than the average cell in a human body. Their small size, flexible fabrication, and high surface-area-to-volume ratio make them ideal systems for drug delivery. Nanoparticles can be made from a variety of materials including metals, polysaccharides, and proteins. Biological protein-based nanoparticles such as silk, keratin, collagen, elastin, corn zein, and soy protein-based nanoparticles are advantageous in having biodegradability, bioavailability, and relatively low cost. Many protein nanoparticles are easy to process and can be modified to achieve desired specifications such as size, morphology, and weight. Protein nanoparticles are used in a variety of settings and are replacing many materials that are not biocompatible and have a negative impact on the environment. Here we attempt to review the literature pertaining to protein-based nanoparticles with a focus on their application in drug delivery and biomedical fields. Additional detail on governing nanoparticle parameters, specific protein nanoparticle applications, and fabrication methods are also provided.

## 1. Introduction

Drug delivery systems are a valuable means of disease treatment and prevention in today’s medicine. Prior to the introduction of drug particle microencapsulation in the 1950s, drug delivery was based on rudimentary practices such as applying poultices or consuming herbal ingredients [1]. These methods, while moderately effective at the time, are inefficient and pose unnecessary health risks. However, progress in the understanding of pharmacokinetics has led to the development of sophisticated and novel methods for administering a variety of therapeutics throughout the body. Now, drug delivery methods allow for controllable drug-release and targeting to improve the safety and efficacy of treatment. To further enhance drug delivery, nanotechnology has begun to be implemented in the field. Specifically, the use of nanoparticles as carriers is an effective strategy to deploy medications to specifically targeted parts of the body [2,3].

Nanoparticles, or microspheres, are ideal drug delivery systems for both controlled and targeted drug delivery. Their sizes typically range between 1–100 nanometers in diameter and can extend to more than 1000 nanometers. However, particle sizes smaller than 200 nm are more preferable for use in nanomedicine due to their ability to traverse micro-capillaries [4]. There are several aspects to consider, such as size and surface charge as examples, before selecting an appropriate nanoparticle material [5,6]. While nanoparticles can be fabricated from synthetic materials, protein-based nanoparticles have received considerable more attention due to their biodegradability and tunable properties [6,7]. Advances in medical technology have also brought about techniques to synthesize protein-based materials that offer improved efficacy and reduced costs compared to synthetic materials [8].

Protein polymers are natural macromolecules derived from plants and animals which makes them an easily obtainable, renewable resource. In addition to their biodegradability and tunable properties, nanoparticles fabricated from protein-based materials are often biocompatible and can be easily processed [9,10]. There are a variety of different protein polymers suitable for nanoparticle-based drug delivery each with their own unique structure-function relationships. In this review, the structure and property relationships of these natural protein-based polymers will be discussed, as well as their methods of preparation. The use of these nanoparticles in medicine will then be reviewed with a focus on their application for nanoparticle-based drug delivery.

## 2. Categories of Protein Materials

**Figure 1:**
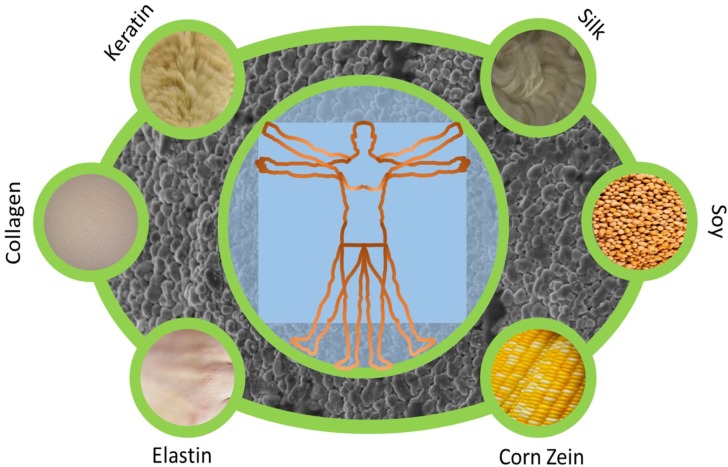
Nanoparticle materials can be fabricated from a variety of protein sources, including silk, keratin, collagen, elastin, soy, and corn zein etc. These proteins can then be processed into particles with unique properties for biomedical applications.

Due to the wide range of applications for protein nanoparticles, there are many types of proteins that are used to create protein nanoparticles. The type of protein polymer required may vary depending on the application. In this review, silk fibroin [11], keratin [12], collagen [13], gelatin [14], elastin [15], corn zein [16], and soy protein [17] will be given particular attention due to their popularity in biomaterials research (Figure 1). However, additional protein polymers such as casein [18], fibrinogen [18], hemoglobin [19], bovine serum albumin [20], gluten [20] have also been used to create nanoparticles.

### 2.1. Silk Fibroin

Silk fibroin protein is among the most popular natural polymers used for the creation of biomaterials due to its acceptance by the US Food and Drug Administration (FDA), low cost, and abundance. Commonly extracted from silk produced by the *Bombyx mori* silkworm, fibroin can be easily isolated after removal of the external sericin protein coating through treatment with sodium carbonate. The resulting fibroin protein is made of semi-crystalline structures comprised of a light and heavy chain [21]. An isoelectric point (IEP) below pH 7 and molecular weight of 83 kDa have been reported for regenerated silk fibroin, but the latter value may vary depending on the extraction procedure and duration of treatment [22,23]. The repetition of amino acids in the pattern (Gly-Ser-Gly-Ala-Gly-Ala)_n_ leads to crystalline beta-sheets that are then stacked in an antiparallel configuration [24,25,26]. This structure gives silk fibroin robust mechanical properties and high tensile strength. The crystallinity and conformation of silk fibroin can also be modulated to allow for high encapsulation of drugs while preserving their pharmaceutical activity [27].

Silk-based nanoparticles have proven effective in the delivery of both hydrophobic and hydrophilic drugs such as indomethacin and aspirin [28] of varying molecular weights, as well as anti-cancer therapeutics such as doxorubicin [11], bioactive molecules including growth factors VEGF ((vascular endothelial growth factor) and BMP-2 (bone morphogenetic protein 2) and Horseradish peroxidase and glucose oxidase enzymes [29], as well as plasmid DNA [30]. Silk-composite nanoparticles have also been fabricated by combining the protein with other biopolymers such as insulin [31], chitosan [32], and albumin [33] and synthetic polymers such as polyvinyl alcohol [34], polylactic acid [35], and polycaprolactone [36]. These approaches allow for a greater degree of tunability that can potentially increase the efficacy of drug delivery.

### 2.2. Keratin

The use of keratin as a biomaterial has been rapidly expanding over the past 40 years because of its abundance, low cost, biocompatibility, and its ability to biodegrade safely [37]. Keratin is a fibrous structural protein with molecular weight of up to 63 kDa and IEP between pH 4.5 and 5 that is derived from the human or animal epidermis and epidermal appendages, such as hair, scales, feathers, and quills in mammals, reptiles, and birds [38,39,40]. The keratin protein is most commonly found in epithelial cells. It is a structural protein that provides the framework for cell-cell adhesion to form a protective layer. Keratin structure is a left-handed alpha-helix which can be coiled together with other keratin proteins to form a polymerized complex. There are three different forms of keratin: α-, β-, and γ-keratins. α-keratins contain intermediate filaments, which are involved in the cytoskeleton, and are mainly found in soft tissues. β-keratins also contain intermediate filaments, but are found in hard tissues, such as scales and nails. γ-keratin is not involved in the structural elements of the cytoskeleton [37].

According to recent studies, keratin-based nanoparticles are effective anticancer drug carriers possessing a degree of tumor targeting ability and controlled drug release [12,41]. Disulfide bonds from cysteine residues and hydrogen bonds from amine groups grant keratin nanoparticles the durability to deliver drugs with high molecular weight to their target location. In addition, keratin is negatively charged allowing positively charged molecules to better adhere to the nanoparticle for more effective transport. The targeting ability of keratin-based nanoparticles is attributed to their pH sensitivity [12,41,42,43]. Keratin-based nanoparticles can respond to changes in pH to release their drug contents accordingly in a controlled release. Due to its intrinsic water stability, keratin is also a desirable support polymer for synthetic nanoparticle composites [43]. Silver nanoparticles coated with keratin are shown to have improved stability in aqueous environments [44]. Keratin is also advantageous for supporting cell adhesion and promoting cellular proliferation [41,45]. Gold nanoparticles coated with keratin are shown to exhibit biocompatibility with improved antibacterial activity [46]. Keratin appears to be an ideal drug carrier which should be investigated further for drug delivery purposes.

### 2.3. Collagen and Gelatin

Collagen is the most abundant biopolymer in the human body [47]. This fibrous protein is a major component of the extracellular matrix and is responsible for maintaining its structure. The majority of collagen is located in connective tissues such as the skin, tendons, and ligaments [48]. Collagen can be divided into two different groups: non-fibrillar and fibrillar, which can be further divided depending on the structure and use. Type 1 of fibrillar collagen is the most common type found in the human body and has a molecular weight in the 100 kDa range. Long, triple helical structures are responsible for strength and flexibility in collagen. This helical structure has high mechanical strength due to a repeating amino acid sequence Gly-X-Y, where “X” and “Y” are commonly proline, hydroxyproline, leucine, or lysine. The individual helical structures, known as tropocollagens, will bind together and form a fibril structure. These fibril structures can then be cross linked together to form suitable cell scaffolds for use in tissue engineering [49].

Due to collagen’s biocompatibility and low antigenicity, collagen-based nanoparticles have been used for the delivery of pharmaceuticals such as theophylline, retinol, tretinoin, and lidocaine [13,50,51]. Collagen is capable of resembling the microenvironment of some tumors allowing collagen nanoparticles to effectively infiltrate the areas and deliver anticancer therapeutics [52]. Physical properties of collagen nanoparticles such as size, surface area, and absorption capacity, are easy to configure [53]. This makes collagen nanoparticles a prime candidate for controlled drug release strategies.

In comparison, gelatin is a biopolymer derived primarily from insoluble Type I collagen through thermal denaturation or disintegration [54]. Like collagen, gelatin has received much attention in the biomedical field due to its biocompatibility and high abundance. Gelatin contains a triple helical structure, similar to collagen, made of repeating amino acids: alanine, glycine, and proline [55]. Depending on the production process, gelatin can be classified as type A or type B and consist of varying molecular weights. Type A gelatin is extracted through an acidic process, while type B is process under alkaline conditions [56]. Type A gelatin is positively charged and has an IEP of approximately pH 9. Conversely, type B gelatin is negatively charged and has an IEP of pH 5 [56]. Tissue engineering scaffolds have thus been made from gelatin as well. Alternatively, gelatin can also be formed into a gel which can be used in the place of thermoplastic polymers [57].

Gelatin is also a favorable nanoparticle material due to its relatively low antigenicity and non-carcinogenic nature [14,58]. Gelatin nanoparticles are extensively used as successful anticancer drug carriers [59] and gene delivery vehicles [60]. Gelatin nanoparticles are able to deliver drugs across the blood brain barrier, which is a semipermeable barrier that is highly studied for drug delivery systems [55]. Gelatin nanoparticles have also safely and efficiently carried *NS2*, a recombinant gene from the hepatitis C virus, without negatively impacting the function of the gene [61]. In addition, gelatin can be blended with other natural polymers to enhance their therapeutic behavior. An alginate-gelatin composite nanoparticle benefitted from an electrostatic bond formed between the two polymers and allowed for a more controlled release of the encapsulated drug, doxorubicin [62]. Utilizing gelatin is both a promising and convenient approach for nanoparticle-based delivery of genes, vaccines, and drugs.

### 2.4. Elastin

Elastin is an important protein found in elastic fibers, specifically in the extracellular matrix. It provides support and elasticity to many structures such as the heart, lungs, skin, and blood vessels with high molecular weight species weighing 130 to 140 kDa [63]. It is insoluble and therefore can retain its shape and insolubility after stretching [8]. However, insoluble proteins are often not biocompatible and are difficult to alter. Through the use of recombinant proteins and peptide synthesis, soluble proteins that have elastin-like properties called elastin-like-peptides (ELP) are able to be produced with tunable molecular weights [64]. These polypeptides are derived from tropoelastin, the building block of elastin. This precursor molecule is vital in the exploitation of ELP’s. ELP’s are able to react to stimuli due to their temperature sensitivity, which induces a phase transition [65]. They can then self-assemble by the process of coacervation into a more ordered structure such as beta-spiral structure [66]. This property, along with their biocompatibility, makes them excellent prospects for biomedical applications. Elastin-based proteins also have the ability to communicate with cells through naturally occurring cellular receptors such as elastin binding protein (EBP) [67]. This receptor can be exploited by using tropoelastin-based polymers to induce or inhibit various cell functions [66].

Elastin, or ELP, nanoparticles have proven effective in delivery of cytokines such as BMP-2 and -14 [68], anticancer therapeutics such as doxorubicin [69], and genes [70]. Their ability to self-assembly when exposed to certain temperatures serves as a mechanism to entrap active substances [15] and achieve controlled drug release [69]. The polymer functionality of ELP nanoparticles can be controlled by using a recombinant fabrication technique [69,70]. This means that variables pertaining to drug release, such as composition and molecular weight, can be tailored for a variety of applications in drug delivery.

### 2.5. Corn Zein

Zein is low molecular weight protein (20 kDa), found within the cytoplasm of corn cell endosperm and is insoluble in water except in the presence of alcohol, urea, alkali, and anionic detergents [71]. The protein has an IEP of pH 6.2 and is a mixture of two different peptides: α zein and β zein [72]. α zein is the most widely used variety due to its abundance [73,74]. Zein has a helical wheel shaped structure with nine homologous units arranged in a non-parallel way with hydrogen bonds stabilizing it. This helical shape gives zein a globular structure similar to insulin and ribonuclease [73]. Zein can be extracted using primary, secondary, and ternary solvents. Primary solvents consist of a compound that dissolves zein in a concentration greater than 10%. Secondary solvents are organic compounds. Ternary solvents are a combination of solvent, water, and alcohol. Zein is commonly used in fibers, adhesives, plastics, ink, chewing gum, and as a preservative coating for some food and pharmaceuticals [74].

Zein nanoparticles are successful drug carriers for encapsulation and controlled release of fat soluble compounds such as α-tocopherol [75,76,77], other proteins [16], vaccines [16,78], and vitamins such as D3 [79,80]. Due to the protein’s hydrophobicity, zein nanoparticles are promising oral drug delivery vehicles able to protect encapsulated contents from harsh acidic environments such as in stomach acid [79]. Zein nanoparticles can also have their properties improved by combining the natural polymer with other substances. For example, sodium caseinate was incorporated with zein nanoparticles to improve particle stability in water [81]. Zein nanoparticles are an attractive drug delivery system due to their high stability in a variety of environments and tunable properties in combination with certain molecules.

### 2.6. Soy

Soy protein is a globular protein isolated from soybeans, known as soy protein isolate, and is one of the most abundant types of plant proteins. The globular structure is comprised of two major subunits, conglycinin and glycinin, which contain all amino acids particularly glutamate, aspartate, and leucine [82]. This structure composition gives soy protein relative stability for long storage life [83] and biocompatibility [84]. When the globular protein is treated with enzymes, soy protein hydrolysates below 1 kDa and between 1 and 5 kDa can be obtained and further processed [85]. In addition, soy protein is biodegradable as it can be digested if consumed. For example, soy protein-based edible films are often used as a wax coating for fruits to preserve their quality [86,87,88]. Soy protein films, scaffolds, and hydrogels have also been applied in tissue engineering for wound healing and transdermal drug delivery [89]. With every amino acid available, soy protein is effective in supporting cellular communication and cell proliferation. The amino acid composition may also attribute to soy protein being used as protection against bacterial infection [83,90].

Soy protein nanoparticles are becoming more popular due to the high abundance and low cost of the protein, as well as its biodegradability and low immunogenicity. The amino acid composition gives soy protein nanoparticles an advantage in encapsulation of highly hydrophobic drugs [17]. Unlike zein, soy protein nanoparticles are soluble in aqueous environments which can be used in different oral drug delivery scenarios. Soy protein isolates are used as a coating in conjunction with other materials either for protection [91] or for physical or chemical surface modification [17,92]. For example, magnetic nanoparticles prepared with soy protein isolate benefit from enhanced functional surface area increasing the loading of enzymes [92]. The protein coating also offers a degree of bioinert behavior to otherwise non-immunogenic nanoparticle materials.

### 2.7. Other Proteins: Casein, Fibrinogen, Hemoglobin, Bovine Serum Albumin, Gluten

Along with the many proteins mentioned above, there are some that will be excluded from this review but are worth mentioning. Casein, fibrinogen, hemoglobin, bovine serum albumin, and gluten are just a few of many. Similar to those previously explained, the use of these proteins depends on their properties and the application’s demands. Casein is very useful in hydrophilic environments since casein is a hydrophilic protein in itself. It is useful for water-based environments since as a microsphere they disperse instead of aggregate [18]. As a micro-/nanosphere, fibrinogen polymerizes when used in conjunction with a serine protease and forms a protein mesh that can be used to cover and treat open wounds or used in vitro for more in depth biomedical applications [93]. Hemoglobin as a micro-/nanoparticle can be used as an oxygen deposit to make oxygen releasing biomaterials [19]. Bovine serum albumin can be used to pack prepared protein particles to aid in protein and drug delivery [94]. Gluten as a microsphere can be used as a drug delivery vehicle that is very effective compared to other widely used proteins [20]. While these proteins are not described in further detail in this review, each protein possesses their own unique advantages when applied in nanoparticle-based drug delivery.

## 3. Fabrication Methods

Due to the necessity of obtaining particles of different sizes, shapes and weights, there are many fabrication methods that are available for the creation of nanoparticles. Fabrication methods will also vary depending on the properties of the individual polymers, such as temperature dependence. Fabrication methods that will be discussed in this review include pH variation, spray-drying, phase separation, milling, rapid laminar jet, and polymer chain collapse. The synthesis of blended protein-based nanoparticles will also be discussed. The advantages and disadvantages of these fabrication methods are summarized in Table 1.

### 3.1. pH Variation

The drug delivery properties of silk fibroin can be modified by changing many factors during nanoparticle synthesis. One of these factors is the pH of the silk fibroin [95]. Particles are made by salting out a fibroin solution with potassium phosphate. The pH of the particles can be controlled depending on what type of potassium phosphate is used in the salting out. Mono potassium phosphate has a pH of 4 and dibasic potassium phosphate has a pH of 9. Silk fibroin particles with a pH of 4 develop silk II rich secondary structures while silk fibroin particles with a pH of 9 developed a silk I rich secondary structure. Particles with the silk II structure or the lower pH are less chemically stable than the particles with a higher pH and the silk I structure. When a positively charged drug is loaded into a negatively charged silk fibroin particle there is a difference in the release depending on the pH of the particle. Particle with the silk II structure and low pH have an increased initial release, whereas the high pH particles have a low release. However, particles at a neutral pH of 7 had an overall increased release over the entire time not just initially.

### 3.2. Spray-Drying

**Figure 2:**
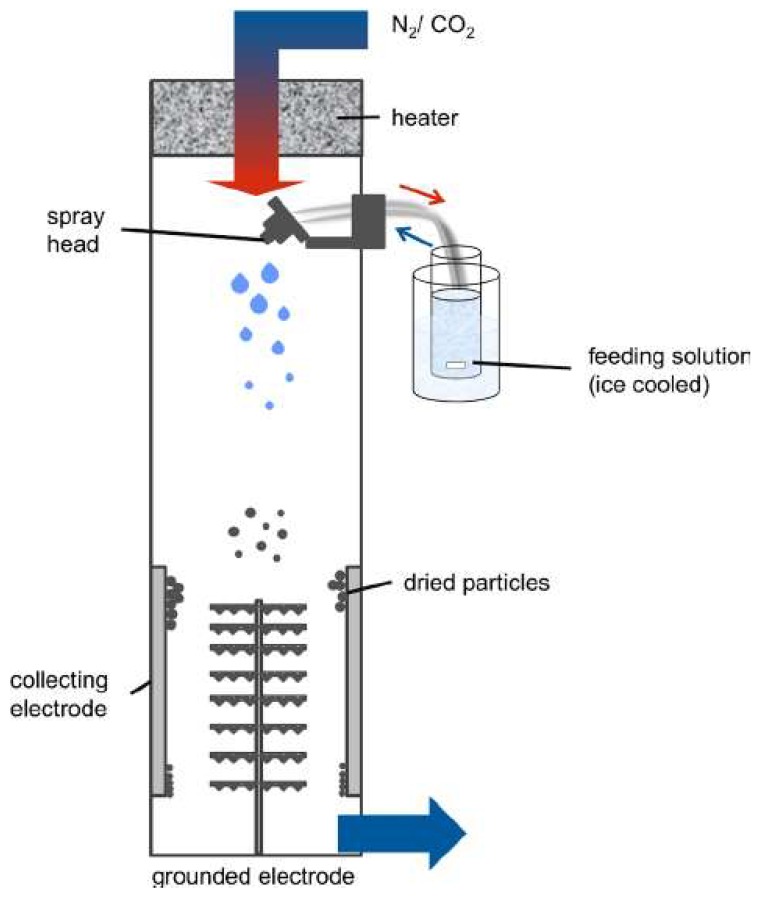
A schematic of a nanoparticle spray-drying system in which the liquid polymer sample is sprayed alongside of heated gas in a chamber that leads to electrodes which are used to collect the charged sprayed nanoparticles. (Reproduced with permission from [102], Copyright Springer Nature, 2015).

Spray-drying is a technique that is used to fabricate nanoparticles from a liquid sample. The liquid sample is sprayed out of a nozzle into a chamber where heated nitrogen and carbon dioxide gas flow in the direction of the spray (Figure 2). In the bottom of the chamber, there are electrodes which are used to collect the nanoparticles. As the sprayed droplets move towards the bottom of the chamber, they become electrostatically charged due to these electrodes. This is a one-step process that is a quick, cost effective method for small scale protein particle production. One application of spray-drying is for use in drug delivery systems due to the ability of hydrophilic drugs to be encapsulated in these spray-dried nanoparticles [96]. This nanoparticle fabrication technique is useful for samples that are heat-sensitive since the solvent evaporation helps maintain the temperature of the nanoparticle droplets. This method of nanoparticle synthesis also gives the user the ability to control the size of the particle that is produced by changing parameters, such as the size of the nozzle and speed at which they are sprayed out [101].

### 3.3. Rapid Laminar Jet

Particles can be also made using a rapid laminar jet method. The feed liquid will contain a certain number of compounds from which the particle can be made. Spherical drops form when a liquid jet discharges from a small opening at laminar flow conditions. This formation behavior of the drops is resulted because of the surface energy and tension of the jet. The liquid spheres will be dispersed in some type of fluid or gas/air depending on the mechanism. Drop size is-based on the length of the jet. For best results the jet length between breakpoints should be five times the diameter of the stream which gives particle sizes of about twice that of the jet. This laminar breakup of the jet is a result of small disturbances. These disturbances must be controlled to preserve uniformity in drop sizes. To control these disturbances a controlled uniform vibration is applied to the jet. Ideally the frequency of the vibration is close to the naturally occurring frequency for laminar breakup. This leads to a clean controlled breakup and uniform drop sizes [97].

### 3.4. Phase Separation

Out of the various methods of protein nanoparticle fabrication, emulsion-solvent evaporation is the most popular. This technique was the first to form polymer nanoparticles [103]. Organic and aqueous phase separations are the backbone of this method. Prepared polymers are placed in an organic solvent. A surfactant is added to the aqueous phase in order to prevent the fusion of emulsion particles [104]. The solution is then subjected to a mixing method such as ultrasonification. Mini-emulsion droplets of polymer are formed. Finally, the solvent is separated. This is often completed by evaporation of the organic phase. The remaining solution contains polymer nanoparticles which can be collected through a centrifuge. This method produces particles in the 50–500 nm size range. Particle size could be controlled by altering the concentration of polymer solution [105]. This technique is extremely popular due to the availability of conjugated polymers [98].

Another method based on separations is the coacervation method. This is commonly referred to simply as phase separation but for the purposes of this paper it is included in this section. This method requires the separation of two liquid solutions. One will contain the protein polymer and the other is a solvent. Through some means of disrupting equilibrium such as the addition of a salt, coacervation is induced. The charges create electrostatic forces which induce the formation of nanoparticles [98].

### 3.5. Milling

**Figure 3:**
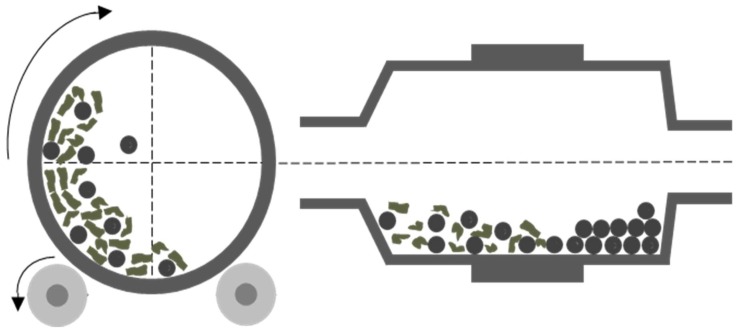
This figure shows the basic mechanism used in high energy ball milling. As the cylinder rotates, the milling balls are accelerated and through physical force fracture the polymer material that is placed in the chamber.

Milling is a fabrication technique for nanoparticles that requires mechanical energy to break down larger particles into fine nanoparticles (Figure 3). This fabrication technique is commonly used for nanoparticles that are to be used in drug delivery [106]. Milling is a cost-effective way to produce nanoparticles in a large-scale production. High energy ball milling involves the subjection of coarse nanoparticles to high energy collisions from the milling balls. Coarse nanoparticle powder is placed in a chamber that contains milling balls and mechanical movement is applied to the cylindrical chamber to accelerate the milling balls which can roll over and collide with the powder. These collisions and other mechanical force from the milling balls causes the coarse nanoparticles to break down into fine nanoparticles. The chamber must be cooled due to the heat energy released from the mechanical energy exerted on the nanoparticles. This fabrication technique allows the user of the system to control the size of the nanoparticles by altering the speed of the rotation of the cylindrical chamber [99].

### 3.6. Polymer Chain Collapse

Single-chain collapse of polymers is a method to produce individual single-chain polymer nanoparticles (SCNP). This method can produce particles in the range of 5–20 nanometers. In addition, intrachain folding produces particles that have great stability compared to other techniques [100]. Control of the precursor chain can also dictate the properties of the nanoparticle, allowing for the production of distinct molecules [107].

There are different varieties of the SCNP method and the type of reaction is dependent on the functional groups involved. However, all the methods benefit more from intramolecular cross-linking rather than intermolecular cross-linking [108]. Homofunctional chain-collapse involves placing a functional group that is likely to bind with itself on the precursor chain and then performing a reaction that couples the functional group [107]. This method often produces particles that are not globular in shape. Instead, heterofunctional coupling is being looked to for improved results. This requires two functional groups which are orthogonally cross-linked. There are many ways to perform the cross-linking in both hetero and homofunctional chain collapses. Data has shown that this method produces nanoparticles with improved spherical shape [109].

### 3.7. Protein Particle Composite

Protein particle composites contain more than one protein or polymer, the addition of which can be used to tune the mechanical and physical properties of a drug delivery vehicle. The method in which they are fabricated depends on the type of particle desired and the differing properties of the additional component. The properties can be a variety of different things such as mechanical properties, electric properties, electromagnetic properties, elasticity, crystallinity, moldability, and many more. When choosing the different type of additional polymer to add, it usually contains an additional characteristic that the main protein does not. Where one protein’s structure may be dependent on pH, adding another material to form a stable complex between the two to withstand a lower pH could make more fabrication methods possible.

For fabrication of these particles, it depends what end product is desired. Any previous or following fabrication method that is described can be used to make composite protein-based particle. The only difference between this method and the others is the fact that a protein composite must be made before or after fabrication. For example, if the particles are going to be fabricated using spray drying, a liquid protein mixture can be made before spray drying is done or individual nanoparticles can be formed and then mixed together to create the same product. If a certain percentage of protein is desired in the final product, it is important that an appropriate fabrication method is selected. Fabrication methods can affect the resulting nature of the particle. Particle behavior depends on their surface composition, geometry, and size among other characteristics. There are many more methods that can be utilized for fabricating protein particles apart from what was described in this review [110].

## 4. Factors to Control Particle Formation

In nanoparticle formation there are many factors that can be controlled to modify drug delivery, such as size, molecular weight, and shape. These factors are mainly determined by the fabrication technique applied but can also be due to the properties of the polymers themselves.

### 4.1. Size

Nanoparticle size can vary depending on the molecular weight of the protein polymer used. Typically, nanoparticle size ranges from 1–100 nanometers but they can extend to 1000 nanometers in diameter [111]. One way to control nanoparticle size is to prevent aggregation of the nanoparticles, which can be done by introducing chemicals that help prevent this aggregation by reducing disulfide bonds or by altering the charge state of the polymers [112]. Other factors related to controlling the size of the nanoparticles vary by the technique used to produce them. With the spray drying nanoparticle manufacturing technique, the size of the particles can be altered by changing the size of the nozzle used to spray the polymeric nanoparticle solution into the drying chamber; the size can also be altered by the speed at which the solution is sprayed [101].

### 4.2. Shape

**Figure 4:**
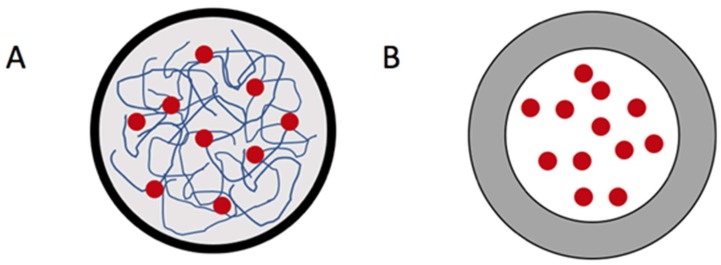
(**A**) A model of protein nanosphere. The drug (red) is within a protein matrix (blue); (**B**) A model of protein nanocapsule. The drug is suspended and encapsulated by a thick protein polymer shell.

There are many different forms of nanoparticles. The two fundamental types are nanospheres and nanocapsules. The main difference between these types are that nanospheres contain a polymer matrix inside, whereas nanocapsules have a shell that separates the encapsulated polymer from the outside environment (Figure 4). Solid lipid nanospheres are being studied as potential drug carriers due to their matrix morphology. This allows for controlled release and protection of the drug [113]. These particles can be formed by subjecting alkyl cyanoacrylates to polymerization in emulsion [114]. An additional method is precipitating polymers that have already been altered [113]. Solid nanospheres may also be formed using microfluidics method. This method is extremely cost efficient and allows for more control of particle features. The solvent volatility can be altered to shape the surface. Variances in flow rate and the architecture of the devices can create different geometries [114].

Nanocapsules are somewhat the opposite of solid nanoparticles. This is based on their hydrophobic and hydrophilic interactions. The counter methods can be applied to form nanocapsules. Adding an oil to the emulsion polymerization results in a core-shell formation [113]. Essentially, the presence or absence of oil dictates which type of nanoparticle will form. Another type of nanoparticle is the Janus nanoparticle. These particles consist of two different sides, each with their own properties. These properties can include hydrophobicity and hydrophilicity. The combination of functionalities allows for stimuli response and unique assemblies. Janus particles can be formed by masking. This process involves protecting one region of the particle while the other is functionalized. The mask is then removed, and the final product is a particle with a dual nature particle [115]. Another process is self-assembly. First block copolymers undergo phase separation. Then specific cross-linking must occur, followed by disassembly of larger structures [116].

### 4.3. Properties of the Protein

When designing a protein-based nanoparticle, it is important to consider how the protein will interact with the encapsulated drug and physiological environment. Ultimately, a protein with the appropriate molecular weight and IEP must be chosen. The molecular weight of the protein used to create the nanoparticle is important to consider since it can affect how much drug can be effectively stored and the particle targeting mechanism in the body. In some instances, a nanoparticle made from a very high or low molecular weight protein can result in lower encapsulation efficiency. A moderate molecular weight protein is often more appropriate and can help to achieve higher encapsulation efficiency [117]. The molecular weight can also contribute to the pathing of the nanoparticle through the body. In addition to molecular weight, the IEP of the protein will affect the stability of the nanoparticle in different environments. At pH near the IEP, nanoparticles may begin to aggregate and decrease in stability [118]. This can inhibit their circulation throughout the body as well as their drug release. Therefore, a protein with the appropriate IEP and molecular weight must be chosen to ensure that nanoparticles withstand certain environments.

## 5. Novel Applications of Protein-Based Nanoparticles

Protein nanoparticles offer a wide range of uses in medicine as both drug delivery vehicles and bioimaging aids.

### 5.1. Bioimaging

Polymer nanoparticles are gaining traction as contenders to replace typical fluorescent dyes. These are used in non-specific and targeted microscopic imaging [119]. In non-specific imaging, these nanoparticles can be used to dye cells. Data demonstrates that phospholipid encapsulated polymer nanoparticles are successful in providing quality fluorescent imaging of cancer cells. These cells displayed no symptoms of toxicity. In addition, it is possible to tune the wavelength emitted by altering the conjugated protein polymer [120]. These properties, along with an increased circulation period, could lead to applications in vivo. In addition, protein nanoparticles have a bright future in targeted cellular imaging. These particles have an increased uptake due to the enhanced permeability and retention of advanced tumors. Near IR light can provide excellent imaging quality when paired with a polymer nanoparticle-based probe due to the previously mentioned properties [121]. Overall, the applications of these particles in the biomedical imaging field are rapidly growing.

To enhance the biocompatibility and cellular uptake of nanodiamonds (ND), Khalid et al. encapsulated the material in silk fibroin nanospheres using a co-flow technique. Due to silk fibroin’s transparency and low background signal, the photoluminescence of the NDs was not diminished. In fact, NDs encapsulated in silk fibroin spheres fluoresced 2–4 times brighter than NDs alone. The 400 to 600 nm spheres were also found to be highly stabile in an aqueous environment, but began to degrade after one week of incubation at 37 °C. When introduced to fibroblast cells in vitro, the intracellular mobility and diffusion of NDs was improved [122]. Li et al. also used silk fibroin to create nanoparticles for bioimaging, through hydrothermal treatment that simply involved heating the protein at 200 °C for 72 h. This procedure produced water-soluble, nitrogen-doped, photoluminescent-polymer-like carbonaceous nanospheres (CNSs) that measured approximately 70 nm in diameter and could easily be isolated through filtration. These nanoparticles exhibited low cytotoxicity when incubated with HeLa cells and fluoresced in the perinuclear regions once ingested. CNSs could also be used to image tissue at a depth of 60 to 120 μm with no blinking and low photobleaching [123]. These studies illustrate the improvements that may result from the incorporation of protein into nanoparticles for bioimaging.

In addition to silk fibroin, gelatin nanoparticles have also been utilized as bioimaging platforms. Liu et al. created gelatin nanocapsules containing gold nanoparticles by denaturing gelatin polypeptides that then absorbed onto citrate-stabilized gold nanoparticles. A thin layer of silica was then used to stabilize these particles that measured approximately 50 nm in diameter and hold promise in Raman-active bioimaging [124]. Gelatin has also been used to coat Cadmium telluride (CdTe) quantum dots (QDs), leading to an improvement in their cytotoxicity and biocompatibility. In this study, Byrne et al. introduced gelatin single- or multi-stranded polypeptides during QD synthesis, to control their growth and nucleation. Functional groups present in the glycine, proline, and 4-hydroxy proline residues of gelatin were then able to interact with the surface of the CdTe QDs, allowing for their coating. When incubated with macrophages, these “jelly dots” were successfully engulfed by the cells, resulting in the illumination of their membranes. When compared to QDs alone, cells exposed to QDs treated with gelatin showed a lower lysosomal pH and cellular permeability, suggesting decreased toxicity of the particles [125]. In both studies, the ability to further functionalize the surface of these particles due to their natural polymer coating, may further enhance their efficacy in bioimaging.

### 5.2. Drug Delivery Vehicle

Protein-based nanoparticles have also found new use as drug delivery vehicles. In addition to their biocompatibility and biodegradability, the surface of protein nanoparticles can be easily functionalized due to their defined primary structure, while charged proteins can facilitate drug loading through electrostatic interactions [6,126]. Such particles can also often be fabricated under mild, aqueous conditions, making them easier and safer to process than ones based on synthetic polymers [95]. The use of natural proteins has also been shown to increase cell retention and reduce the effects of toxic byproducts produced during degradation [127].

One such protein used to create nanoparticles for drug delivery is corn zein. Due to its hydrophobic nature, this protein is especially suited for the prolonged, controlled release of pharmaceuticals. Lai et al. noted this effect when they used the protein to create nanoparticles loaded with the chemotherapeutic agent, 5-Fluorouracil (5-FU). These particles were synthesized using a standard phase separation procedure and measured approximately 115 nm in diameter. The corn zein particles were able to encapsulate 5-FU at an efficiency of up to 56.7% which was then released after an initial burst of 22.4%. When injected into mice, the nanoparticles remained in circulation for 24 h before localizing to the liver due to their high molecular weight [127]. Corn zein nanoparticles have also been used for the controlled release of vitamin D3 [80], therapeutic proteins such as catalase and superoxide dismutase [128], and anti-diabetic drugs [126]. In the latter study, Xu et al. developed hollow zein-based nanoparticles through a two-step procedure. This fabrication technique began with the mixing of corn zein and sodium citrate ethanol-based solutions. The zein polymer than aggregated around the sodium citrate crystals, resulting in the formation of particles with a sodium citrate core and zein shell. To create hollow particles, the core-shell particles were added to water, leading to the dissolution of the sodium citrate core. The resulting hollow nanoparticles measured less than 100 nm in diameter and were able to encapsulate 30% more drug compared to solid zein particles. This drug was then released in a more sustained, prolonged manner over 200 h. When incubated with 3T3 fibroblast cells, the particles were also successfully internalized by cells without effecting their viability [126].

Other plant-based proteins such as soy protein have also been used to create nanoparticles for the controlled release of nutrients and pharmaceuticals. Due to soy’s balanced composition of nonpolar and polar residues, it can act as a versatile carrier by storing drugs with various functional groups. Using a desolvation method and a glutaraldehyde crosslinker, particles measuring between 200 and 300 nm in diameter were fabricated and loaded with curcumin. Curcumin was then released with an initial burst of over 50% within the first 1.5 h, but continued to be released in a more controlled manner over the next 8 h [17]. Although cell studies were not conducted, the established biocompatibility of soy suggests that the particles were act as suitable drug delivery vehicles [84].

Negatively charged proteins such as keratin have also been used to fabricate nanoparticles that are able to incorporate drugs through electrostatic absorption. When prepared through ionic gelation, keratin particles allowed for the long-term and controlled release of the model drug chlorhexidine (CHX). This fabrication technique involved the dropwise addition of a CHX solution to one containing keratin. Negatively charged carboxylate groups on the outside of keratin aggregates attracted the drug, allowing for its retention. CHX was then gradually released over 140 h in a pH-sensitive manner, with greater release occurring at acidic and neutral pH [12]. Cheng et al. also created keratin-based nanoparticles consisting of varying ratios of the oxidized (keratose or KOS) and reduced (kerateine or KTN) forms of the protein. These particles were created using an ultrasonic dispersion technique and measured between 345 and 400 nm, with decreasing diameter upon the addition of more KOS. The addition of KOS also resulted in an increased release rate of the model drug, Amoxicillin (AMO). The nanoparticles were found to be mucoadhesive due to the electrostatic interaction and disulfide bonding between gastric mucin and KTN, and hydrogen binding with KOS. These results suggest that keratin-based nanoparticles may be an ideal carrier for mucoadhesive drug delivery [42].

## 6. Conclusions

Protein polymer and protein composite materials are becoming more accepted in the realm of nanoparticle drug delivery. Their properties are ideal for drug delivery systems and show promise in improving controlled release or targeting delivery mechanisms. Natural protein polymer is relatively cheap, easy to process, and renewable which makes it an attractive material from an economic perspective. The primary advantages that protein-based nanoparticles have over synthetic materials is their biodegradability and biocompatibility. Minimizing the host immune response is an important aspect determining the success of a drug delivery operation. The natural degradation of these protein polymers reduces accumulation of particle byproduct which is also better for the human health. 

This review focused on the properties of protein materials, such as silk fibroin, keratin, and elastin, and their usage in nanoparticle drug delivery and biomedical applications. There are a variety of processing methods for protein-based nanoparticles which can tune their resulting properties for more specific applications. While there are still challenges to overcome, there is an increasing demand for biocompatible protein nanoparticles in the medical field. To overcome these challenges, future work involving protein-based nanoparticles must focus on the development of large scale production techniques that allow these particles to be manufactured in a commercially attractive manner. Functionalized particles capable of targeting specific areas of the body are also likely to be developed to limit off-target effects. With the development of new pharmaceuticals, the fabrication and characteristics of protein nanoparticles must also adapt to provide ideal vehicles for drug delivery. As these new studies emerge and the functionality of these protein materials is improved, the more opportunities there will be for effective disease treatment in the future.

## Figures and Tables

**Table 1:** Advantages and disadvantaged of the common protein-based nanoparticle fabrication methods.

Method	Advantages	Disadvantages
pH Variation [95]	Control for particle size Control secondary structure of protein Control for zeta potential Produces chemically and physically stable particles Experimentally simple	Post-fabrication drug loading is required Limited to small scale production
Spray-drying [96]	Cost effective Experimentally simple Easily encapsulate hydrophilic drugs Useful for heat-sensitive samples Control for particle size	Limited to small scale production Challenging to incorporate hydrophobic drugs
Rapid Laminar Jet [97]	Control for particle size Production of uniform particles Production of strong, stable particles	Possibility of coalescence Many parameters must be controlled for
Phase Separation [98]	Specialized equipment is not required Particle size can be controlled by adjusting protein concentration Uniform particles are produced	Particle sizes are limited to 50–500 nm in diameter Organic solvents are required Limited to small scale production
Milling [99]	Cost effective Large scale production is possible Control of nanoparticle size Experimentally simple	Heat is released during the process requiring chamber to be cooled Little control over nanoparticle shape Nanoparticles must be coarse
Polymer Chain Collapse [100]	Properties of the nanoparticle can be easily controlled by selection of the precursor chain	Particle size is limited to 5–20 nm in diameter
	Production of particles with high stability Particles with improved spherical shape are produced	Side reaction may be difficult to control
